# The use of fibrin matrix-mixed gel-type autologous chondrocyte implantation in the treatment for osteochondral lesions of the talus

**DOI:** 10.1007/s00167-012-2096-1

**Published:** 2012-06-30

**Authors:** Kyung Tai Lee, Jin Su Kim, Ki Won Young, Young Koo Lee, Young Uk Park, Yong Hoon Kim, Hun Ki Cho

**Affiliations:** 1 Foot and Ankle Clinic, KT Lee’s Orthopedic Hospital, Seoul, Korea; 2 Surgery of Foot and Ankle, Eulji Medical Center, College of Medicine, Eulji University, 14 Hangeulbiseok-Gil, Nowon-gu, Seoul, 139-711 Korea; 3 Department of Orthopedic Surgery, Bucheon Soon Chun Hyang University Hospital, College of Medicine, Soon Chun Hyang University, Bucheon, Korea

**Keywords:** Talus, Osteochondral lesion, Autologous chondrocyte implantation, Arthroscopy, Donor

## Abstract

**Purpose:**

This study assessed the clinical results and second-look arthroscopy after fibrin matrix-mixed gel-type autologous chondrocyte implantation to treat osteochondral lesions of the talus.

**Methods:**

Chondrocytes were harvested from the cuboid surface of the calcaneus in 38 patients and cultured, and gel-type autologous chondrocyte implantation was performed with or without medial malleolar osteotomy. Preoperative American orthopedic foot and ankle society ankle-hind foot scores, visual analogue score, Hannover scoring system and subjective satisfaction were investigated, and the comparison of arthroscopic results (36/38, 94.7 %) and MRI investigation of chondral recovery was performed. Direct tenderness and relationship to the active daily life of the donor site was evaluated.

**Results:**

The preoperative mean ankle–hind foot scores (71 ± 14) and Hannover scoring system (65 ± 10) had increased to 91 ± 12 and 93 ± 14, respectively, at 24-month follow-up (*p* < 0.0001), and the preoperative visual analogue score of 58 mm had decreased to 21 mm (*p* < 0.0001). Regarding subjective satisfaction, 34 cases (89.5 %) reported excellent, good or fair. Chondral regeneration was analysed by second-look arthroscopy and MRI. Complications included one non-union and two delayed-unions of the osteotomy sites, and 9 ankles (9/31, 29.0 %) sustained damaged medial malleolar cartilage due to osteotomy. Marked symptoms at the biopsy site did not adversely affect the patient’s active daily life.

**Conclusions:**

Fibrin matrix-mixed gel-type autologous chondrocyte implantation using the cuboid surface of the calcaneus as a donor can be used for treating osteochondral lesions of the talus.

**Level of evidence:**

Therapeutic study, prospective case series, Level IV.

## Introduction

In cases of osteochondral lesions of the talus, there are several ways to reconstruct the hyaline cartilage after osteochondral debridement, *that is, autologous chondrocyte implantation (ACI)*, mesenchymal stem cell implantation and osteochondral graft [4, 23, 29]. For ACI, chondrocytes are first collected and then cultured for cell proliferation. The proliferating cells are then inserted into the defective sites. It has been reported that even if a two-stage operation is necessary, ACI may be able to overcome the shortcomings of osteochondral grafts and has been highly successful for treating osteochondral lesions of the talus [2, 3, 10, 28, 38]. Currently, second-generation *matrix*-*induced* autologous chondrocyte implantation used for osteochondral lesions avoids this potential problem by using a collagen membrane for the cell matrix on which chondrocytes are seeded and cut to the correct size and shape of the defect [25, 32, 37]. This technique could make treatment easier, and there have been successful results using this technique for treating osteochondral lesions [5, 7, 34]. The present study introduced a matrix-induced ACI with *mixed thrombin and fibrin in gel form for the cell matrix (fibrin matrix*-*mixed gel*-*type ACI)* in the treatment for osteochondral lesions of the talus [16, 20]. The gel-type ACI technique is easier to perform than conventional or other second-generation ACI. The mixture of fibrin, thrombin matrix and chondrocytes is injected into the osteochondral lesion without being affected by the size, shape or depth of the defective area, and the time for the gel to set is a few minutes. In fibrin matrix-mixed gel-type ACI, fibrin–thrombin components support chondrocyte proliferation and chondrocyte migration, and implantation of autologous ‘chondrocyte-fibrin’ constructions resulted in more successful hyaline-like cartilage regeneration than that obtained by ACI in an animal study [15, 26].

Gel-type ACI was performed at the medial side of the talus, and second-look arthroscopy combined with hardware removal was possible to explore the cartilage regeneration in 36 patients, as it is possible to perform a large number of second-look arthroscopy after ACI. The purpose of this study was to investigate the use of fibrin matrix-mixed gel-type autologous chondrocyte implantation and second-look arthroscopy in the treatment for osteochondral lesions of the talus.

## Materials and methods

Thirty-eight patients were selected with a cartilage defect in their ankle joint, and who were unresponsive to non-surgical treatments or bone marrow stimulation techniques (10 cases) or osteochondral plug transplantation (one case) between 2006 and 2010 were included in this study (Table 1). Institutional Review Board approval was obtained to conduct a prospective evaluation of the gel-type ACI. All patients gave their informed consent prior to their inclusion in this study. Patients with generalized osteoarthritic changes or with tibiotalar malalignment at the ankle were excluded. Preoperatively, osteochondral lesions were diagnosed by physical examination and magnetic resonance imaging (MRI). Conservative treatment was initially performed: on the first month, medicated with non-steroid anti-inflammatory drugs and rest with or without removable ankle brace and physical therapy, and on the second month, with added rehabilitation including peroneal strengthening and intra-articular injections of hyaluronic acid three times per week. However, if there was no specific improvement after conservative treatment regardless of stage, gel-type ACI was considered. The mean duration of symptoms or period after previous surgical treatment was 10 months.

**Table 1:** Demographic data of patients receiving the gel-type autologous chondrocyte implantation

Male/female	33 (87 %)/5 (13 %)
Average patient age (years old)	35 ± 11
Average height (cm)	172 ± 7
Average body weight (kg)	74 ± 10
Size of osteochondral lesion of talus	
Average dimension (mm^2^)	194 ± 125
Average width (mm)	12 ± 5
Average length (mm)	16 ± 5
Location of osteochondral lesion of talus	
Medial (need a medial malleolar osteotomy)	31 (85 %)
Lateral	7 (15 %)
Previous operative history	11 (29 %)
Combined instability (lateral reconstruction)	4 (11 %)
Average conservative treatment period (months)	10 ± 7

### Chondrocyte biopsy

**Fig. 1:**
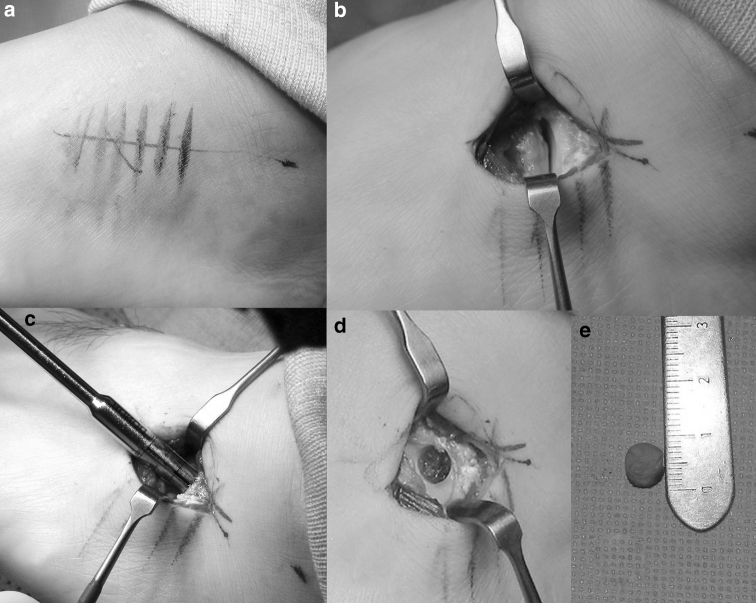
An osteochondral plug harvest. A skin incision approximately 4–5 cm in length was made on the calcaneocuboid joint (**a**) and exposed the joint by dissecting the extensor digitorum brevis and identifying the sural nerve (**b**). A 5-mm-thick cartilage plug was harvested from the superior corner of cuboid surface of the calcaneus using a 7-mm punch at the forefoot supination position (**c**, **d**). The harvested chondrocytes were transplanted onto the culture media and transported to the cell culture laboratory (**e**)

To collect the chondrocytes used to culture autologous chondrocytes, the operation was performed under a foot and ankle block. The calcaneocuboid joint was palpated, and a skin incision approximately 4 cm in length was made on the joint, and the calcaneocuboid joint was exposed by dissecting the extensor digitorum brevis and identifying the sural nerve. A 5-mm-thick cartilage plug was harvested from the superior corner of the cuboid surface of the calcaneus using a 7-mm punch at the forefoot supination position. The cartilage plug was transported to the cell culture laboratory (Sewon Cellontech Co., Ltd., Seoul, South Korea) (Fig. 1). The harvested chondrocytes were transplanted onto the culture media, and a 2D-culture technique was used to make a subculture. When a sufficient number of cells were attained, (approximately 1.2 × 10^7^ cells per vial) and the viability was more than 80 %, the second surgery was scheduled. This usually occurred within the fifth week. Post-operatively, the wound closed and was compressed. Weight bearing was allowed, and the patients were then discharged the following day.

### Chondrocyte implantation

**Fig. 2:**
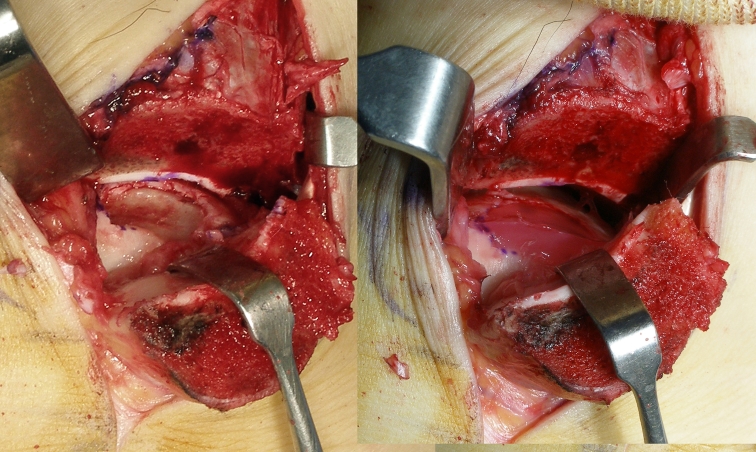
*Left picture* shows the chondral defect on the talus after cartilage debridement. *Right picture* shows a hardened gel form 5 min after injecting a mixture of chondrocytes and fibrin gel matrix into the osteochondral lesion located in the posteromedial area of the talus exposed after performing the medial malleolus osteotomy

The second stage of the gel-type ACI procedure was performed under spinal anaesthesia. If the osteochondral lesion was located on the medial talus, a skin incision was made on the mid-line of the medial malleolus, and the posterior tibial tendon and anterior ankle joint were exposed. The medial malleolus was predrilled with two holes to allow for anatomic reduction at the end of the procedure. Oblique medial malleolar osteotomy was performed under fluoroscopy. The cartilage lesion of the medial talus was exposed by retracting the medial malleolar fragment. The location was determined by a novel, 9-zone anatomical grid system [9]. Dimensions of the osteochondral lesion were determined by multiplying the length and width of the long axis of the defect, and the macroscopic findings were checked according to the Mintz staging system [24] and are applicable to the secondary look arthroscopic findings. The osteochondral lesion was then removed using a number 11 blade or curette. Two to four hole posts, each with a 2–3 mm depth, were made on the osteochondral bed using a 2.7-mm drill bit to stabilize the chondrocyte gel implant. Even if an osteochondral lesion with a cystic lesion was found on MRI, the osteochondral fragment was divided without bone graft or deep curettage. Implantation was then performed by injecting fibrin, thrombin and chondrocytes (Chondron^®^, Sewon Cellontech Co., Ltd., Seoul, South Korea). In this procedure, 2 cc of a liquid mixture containing approximately 1.2 × 10^7^ cells per millilitre was injected. Initially, it was mixed with nutrients needed for the initial survival of the implanted cells. The liquid-type chondrocyte mixture was injected to the defect, and the gel matrix hardened in around 5 min. After completing implantation, the peripheral areas were trimmed and irrigation was performed (Fig. 2). Two 4-mm cannulated screws were fixed for compression without torsional deformity, and an additional 4-mm cannulated screw was fixed parallel to the surface of the ankle joint. The post-operative wound was closed, and a plaster splint was applied with non-weight bearing. When the osteochondral lesion was located on the lateral talus, the anterolateral ankle mini-arthrotomy was used with ankle-placing plantar flexion.

The post-operative ankle was immobilized for 6 weeks with a cast and was followed by a removable ankle brace for 3 months. If radiological union were obtained, then it would permit full weight bearing.

### Evaluations

American orthopedic foot and ankle society ankle-hindfoot (AOFAS) scores [17], visual analogue score (VAS) and Hannover scoring system for the ankle (HSS) [35] were evaluated. The differences were calculated between the preoperative and post-operative scores at 6, 12 and 24 months following surgery, and the degrees in improvement were compared.

The degree of satisfaction was evaluated according to the patient’s own evaluation using a 5-point scale (excellent, good, fair, same and poor). With this scale, excellent, good and fair were considered positive responses. A survey was conducted to determine whether the donor site at the cuboid surface of the calcaneus influenced the function of the ankle joint or caused discomfort in patient performance of daily activities.

**Fig. 3:**
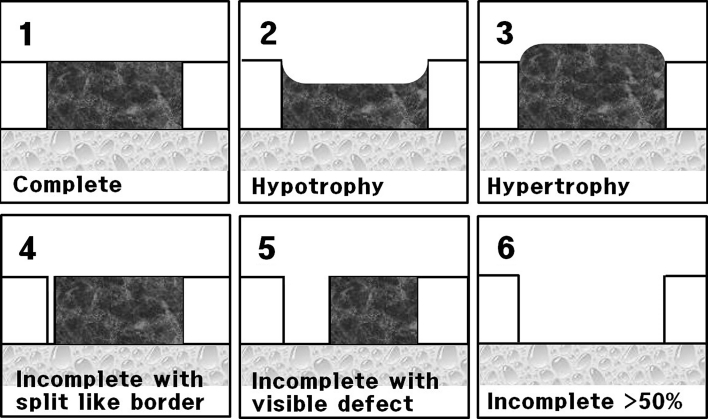
The cartilage regeneration was classified after fibrin- and thrombin-mixed matrix autologous chondrocyte implantation using a 6-point scale: complete healing of the cartilage; complete healing but hypotrophic; complete healing but hypertrophic; incomplete healing with a split-like border; incomplete with visible defect; and more than half of the defect remaining

Second-look arthroscopy and hardware removal were performed 12 months after surgery. The osteochondral lesion was evaluated using the following Mintz grading system for the comparison of the preoperative and post-operative status: 0, normal cartilage; 1, abnormal signal but intact; 2, fibrillation or fissures not extending to bone; 3, flap present or subchondral exposed; 4, loose undisplaced fragment; and 5, displaced fragment. The post-operative regeneration was classified according to the rate of regeneration. The regeneration of cartilage [21] was classified using a 6-point scale as shown in Fig. 3. The International Cartilage Repair System (ICRS) visual score was retrospectively checked with arthroscopic videotapes or photographs of the index procedures [6]. In addition, the possibility of cartilage damage such as synovitis, fibrillation and fibrosis around medial ankle joint caused by the medial malleolar osteotomy was examined.

Preoperative MRI was checked for diagnosis and classification of the initial status of subchondral bone, and 35 patients (92 %) underwent MRI evaluation with Anderson’s modified MRI-based classification system at the post-operative follow-up at 24 months [1].

Histological evaluation was performed in two of these 36 patients. Haematoxylin–eosin, safranin O, alcian blue and Masson’s trichrome and immunostaining for collagen type I and II were performed.

### Statistical analysis

The difference between the preoperative and post-operative AOFAS, VAS and HSS was determined using the paired *t* test. In addition, arthroscopic repair results were compared (ICRS repair category, Mintz grades and 6-point scale rate of regeneration) with AOFAS, HSS, VAS scores using an independent sample *t* test, one-way ANOVA, chi-square test and Pearson’s correlation test. A *p* value <0.05 was considered statistically significant (SPSS 16.0 for windows, Chicago, Illinois).

## Results

The average AOFAS score was 71 ± 14 (min. 31; max. 90) before surgery, 88 ± 8 (min. 72; max. 100) 12 months after the implantation and 91 ± 12 (min. 48; max. 100) at the 24-month follow-up visit. AOFAS scores were significantly improved at 12 months and 24 months after the implantation (*p* < 0.0001).

The average VAS was 58 ± 22 mm (min. 13; max. 100) at the screening, 31 ± 19 mm (min. 0; max. 71) 6 months after the implantation, 26 ± 24 mm (min. 0; max. 82) 12 months after implantation and 21 ± 23 mm (min. 0; max. 85) at the 24 months visit. The VAS was significantly decreased at 6 months, 12 months and 24 months after implantation (*p* < 0.0001).

The average preoperative HSS score was 65 ± 10 (min. 47; max. 92). The HSS score had increased to 88 ± 12 (min. 61; max. 104) 12 months after the implantation and further to 93 ± 14 (min. 57; max. 104) at the 24-month visit. The HSS scores were significantly increased at 12 and 24 months after the implantation (*p* < 0.0001).

The satisfaction of patients was scored as excellent/good/fair/same/poor, and the patient numbers for each category were 14/12/8/2/2 (37/31/21/5/5 %), respectively, at the 24-month visit. Patients mostly described positive satisfaction with the technique as excellent, good and fair scores were scored by 34/38 patients (90 %). At the 24-month visit, all study subjects reported that any pain or discomfort they felt at the donor site did not affect the performance of their daily activities, nor did they influence the functional scores of their ankle joints.

**Fig. 4:**
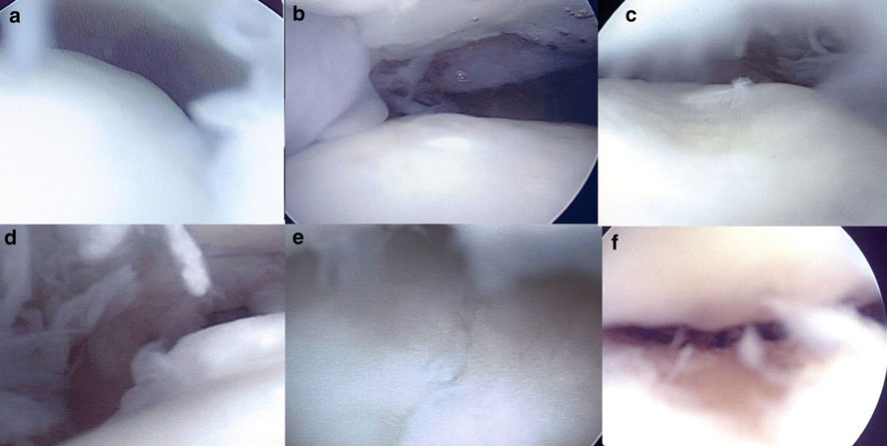
Arthroscopy was performed 1 year after the fibrin-mixed gel-type autologous chondrocyte implantation with medial malleolar osteotomy to observe the results after surgery. The arthroscopic findings of the talus were shown in 7 patients (19.4 %) who had healed with complete marginal integration (**a**, **b**), seven patients (19.4 %) had healed with complete marginal integration, but had hypotrophic cartilage regeneration (**c**), eight patients (22.2 %) had complete marginal integration but had hypertrophic cartilage regeneration (**d**), eleven patients (30.5 %) had healed with incomplete regeneration with a split-like border at the defect marginal site (**e**), and three patients (8.3 %) had an incomplete healing with a visible defect less than 50 % (**f**). No patient had incomplete regeneration of more than 50 % of the defect area. Fourteen of the 36 lesions (38.9 %, more than Mintz grade 3, split border, visible defect) had incompletely covered cartilage at second-look arthroscopy

**Fig. 5:**
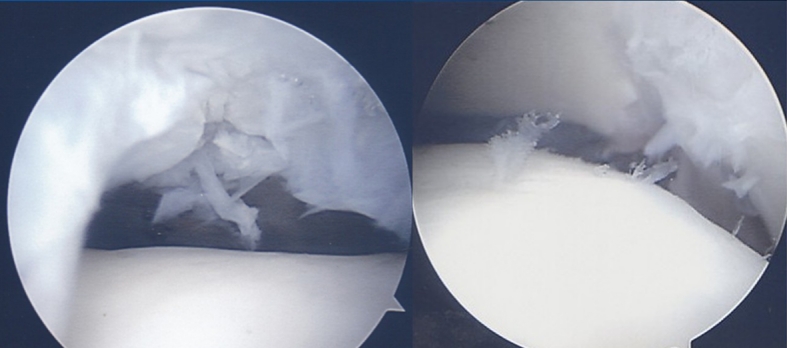
In this study, 9 patients (9/31, 29.0 %) had damaged medial malleolar cartilage caused by the medial malleolar oblique osteotomy. Therefore, medial malleolar osteotomy can cause morbidity

Second-look arthroscopy (36/38 patients, 95 %) and hardware removal (30/31 medial lesions) were performed 12 months after the implantation including the delayed and the non-union patients. By the Mintz grading system, the grade checked at intra-operative period and second-look arthroscopy at 12 months after implantation is shown in Table 2. Most of the flap and loose fragment lesions had disappeared by this time. The arthroscopic findings using a 6-point scale assessed the regeneration macroscopically, as shown in Figs. 3 and 4. The ICRS category was normal (ICRS visual score, 12) in five patients (14 %), nearly normal (ICRS visual score, 11–8) in 22 patients (61 %), abnormal (ICRS visual score, 7–4) in seven patients (25 %) and severe abnormal (ICRS visual score, 0–3) in zero patients. Twenty-seven of the 36 lesions (75 %) were of grade I or II overall. The mean post-operative HSS scores of the ICRS repair category were as follows: 95 for normal or nearly normal and 85 for abnormal, and these scores were significantly different (*p* = 0.047). The mean post-operative VAS of the ICRS category was as follows: 18 for normal or nearly normal and 29 for abnormal, and these scores were not significantly different (n.s.). The mean post-operative AOFAS scores of ICRS categories were as follows: 91 for normal or nearly normal and 91 for abnormal, and these scores were not significantly different (n.s.). Nine ankles (9/31, 29 %) had damaged medial malleolar cartilage caused by the medial malleolar oblique osteotomy (Fig. 5).

**Table 2:** Second-look arthroscopy (36/38 patients, 94.7 %) was performed 12 months after fibrin matrix gel-type implantation

Grade	Cartilage description	Intra-operative	Second-look arthroscopy
Grade 0	Normal	0	0
Grade 1	Abnormal signal but intact	0	6
Grade 2	Fibrillation, fissuring	1	21
Grade 3	Flap, subchondral exposure	13	7
Grade 4	Loose, undisplaced	14	1
Grade 5	Displaced fragment	10	1
Total no		38	36

The Mintz grade checked at intra-operative period and second-look arthroscopy at 12 months after implantation

**Fig. 6:**
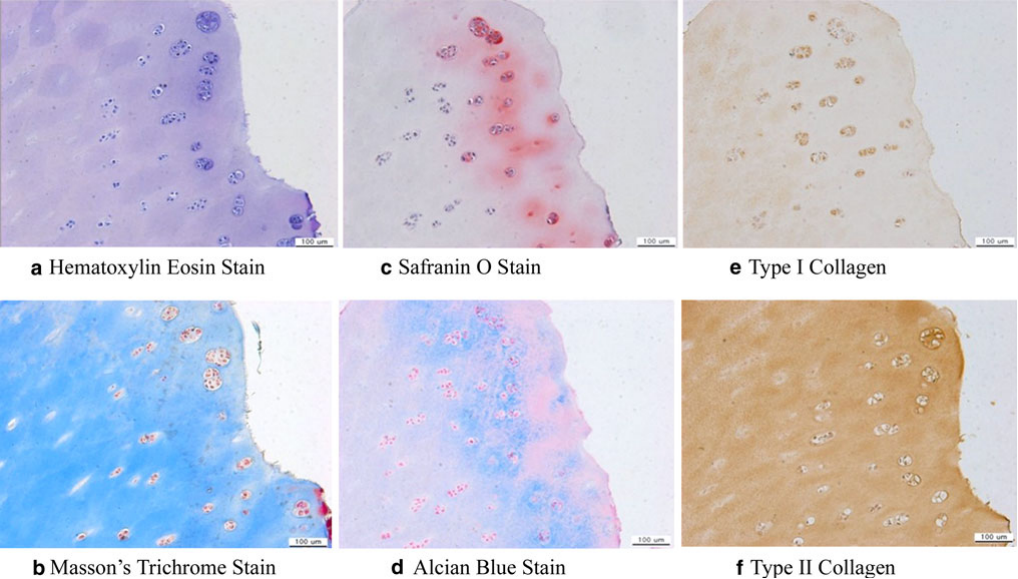
Histological analysis of second-look biopsy harvested from the patient treated with fibrin-mixed gel-type autologous chondrocyte implantation. Sections were cut from second-look biopsies. The sections were stained with haematoxylin and eosin and Masson’s trichrome for cell distribution and collagen in general (**a**, **b**) and with safranin O and alcian blue for glucosaminoglycan distribution (**c**, **d**). The sections were also immunostained using collagen type-specific antibodies for type I collagen and type II collagen distribution (**e**, **f**). The regeneration of hyaline cartilage after fibrin-mixed gel-type autologous chondrocyte implantation was confirmed

Other significant correlations were not found between age, gender, height, weight, medial malleolar osteotomy, Mintz grading, 6-scale rate of regeneration and clinical scores. Table 3 shows modified Anderson’s MRI-based classification of the OCL patients. The arthroscopic specimens obtained from the two patients underwent histological analysis, and the second-look biopsies showed well-developed glucosaminoglycan and type II collagen. Collagen was found to be actively biosynthesized in the cell-implanted sites, and the type of the collagen was found to be mostly type II rather than type I. In addition, glucosaminoglycan was significantly accumulated by the implanted cells, as shown by the alcian blue staining results (Fig. 6).

**Table 3:** Modified Anderson’s MRI-based classification was checked at initial and 24-month visit

Stage	Characteristics	Initial MRI	Follow-up MRI (24-month visit)
Stage I	Marrow oedema	4 (11 %)	2 (6 %)
Stage IIA	Irregular subchondral bone plate	8 (21 %)	28 (80 %)
Stage IIB	Formation of subchondral cyst	6 (16 %)	3 (9 %)
Stage IIC	Incomplete separation of fragment	11 (29 %)	1 (3 %)
Stage III	Unattached, undisplaced fragment with synovial fluid around fragment	3 (8 %)	1 (3 %)
Stage IV	Displaced fragment	6 (16 %)	0 (0 %)
Total number		38	35

Most MRI findings were improved to a higher stage. However, irregular subchondral bone plate, stage IIA observed in 28 ankles, may have been caused by drilling for delamination after implantation. On the follow-up MRI, 10 cases of irregular bone marrow oedema and three subchondral cysts were observed. The three subchondral cysts spontaneously healed

Complications included two patients with delayed non-union which occurred after the medial malleolar osteotomy at 6 and 8 months, respectively, following surgery. Two extracorporeal shockwave therapies were performed to induce bone union. Another non-union patient needed an autogenous iliac bone graft and refixation to make the union.

## Discussion

The most important finding of the present study was that second-generation gel-type ACI performed using a fibrin and thrombin matrix significantly increased AOFAS scores from 71 to 91 at the 24-month follow-up, and cartilage regeneration was observed via arthroscopy follow-up, even though most lesions were medial. Specifically, this study evaluated the cartilage repair with arthroscopic findings by ICRS grade, Mintz scale and 6-point rate of regeneration in this study. Lee et al. [19] reported the first case series with more than 20 patients using second-look arthroscopic objective evaluation and found that 60 % ankles in their study were graded in the normal and nearly normal ICRS category after microfracture and osteochondral lesions. Gianni et al. [10] reported patients with one normal and two nearly normal ICRS category at second-look arthroscopy. In this study, second-look arthroscopic findings showed that 75 % of osteochondral lesions achieved grading in the normal or nearly normal ICRS category (Table 4).

**Table 4:** In this study, second-look arthroscopic findings showed 75 % (36/38) of osteochondral lesions of the talus achieved international cartilage repair system category normal or nearly normal

ICRS category	Normal	Nearly normal	Abnormal	Severe abnormal	Second look/total no.
Microfracture Lee et al. [19] AJSM	12 (60 %)		8 (40 %)	0	20/20
ACI (Hyalograft C patch) Gianni et al. [12] AJSM	1 (33 %)	2 (67 %)	0	0	3/46
Gel-type ACI (Chondron) Present study	5 (15 %)	32 (67 %)	9 (27 %)	0	36/38

These results were better than the results achieved by microfracture

Other reported studies did not describe the cartilage regeneration in more detail. Lee et al. [18] reported second-look arthroscopy of 16 ankles and revealed consistency of the osteochondral grafts and congruity between grafts and native cartilage in 14 (88 %), and a softening or fissuring of the osteochondral graft in two. Nam et al. [27] reported there was no progression of degenerative changes with 10 cases of second look arthroscopy, and synovial inflammation was absent. The osteotomy site healed with no observed joint incongruity or cartilage fraying. Two patients (20 %) had an apparent overgrowth of repair tissue. In the current study, however, nine patients’ ankles (9/31, 29 %) had damaged medial malleolar cartilage caused by the medial malleolar oblique osteotomy including two delayed union and one non-union. Medial malleolar osteotomy can cause cartilage damage at the osteotomy site and morbidity even if it did not significantly decrease the clinical scores. The cause of no clinical significance should be deteriorated by both medial osteochondral lesions with medial osteotomy. In addition, the use of medial malleolar osteotomy sometimes causes delayed bone union or non-union. The osteotomy line should be made on the convex point of the shoulder lesion, and the thinnest possible saw blade should be used to avoid a gap or mismatching of the both fragments. If the osteotomy configuration was made as a chevron or right angle, the fix would be more stable; however, it would be inevitable that the articular side would suffer injury. Therefore, the use of three-screw fixation may be beneficial to reduce translation of bony fragments that can occur with the two-screw fixation method [29, 39]. Thus, two 4–0-mm cannulated screws are placed on an acute angle along the osteotomy line, and one 4–0-mm cannulated screw is placed parallel to the joint line. The third screw’s function is to complete the anti-axial rotation force to add to the other two screws compression force between both osteotomized fragments. Critical to all methods of osteotomy is a precise reduction and fixation to avoid fibrous non-union or malunion. The disturbing of a 4–0-mm cannulated screw’s shaft diameter was not enough for non-union of osteotomized medial malleolus. In a recent study, hypertrophy and delamination were most commonly seen after periosteal ACI (first generation), and arthrofibrosis was most commonly seen after arthrotomy-based ACI (use of a collagen membrane cover, a second-generation technique). In addition, all-arthroscopic, second-generation approaches have reduced the failure, complication and re-operation rate of ACI [14]. However, malleolar osteotomy could increase the morbidity after treating the osteochondral lesion of the talus. Therefore, this study suggests the need for all arthroscopic techniques to treat medial osteochondral lesions of the talus.

The non-weight-bearing cartilage of the knee joint is used more often than the cartilage of the ankle joint as a donor area for the chondrocyte biopsy; however, it has been reported that when the cartilage of the knee joint is used, functional decrement of the knee can occur and creates two separate painful areas, the ankle and knee joints [13, 25, 31, 38]. Whittaker et al. [38] reported that the Lysholm knee score in seven of their ten study patients decreased by 15 % when knee joint cartilage was used as the donor site. Hangody et al. [13] reported that morbidity of the donor site was seen in 3 % of their study patients after ACI. However, they also reported knee joint instability, pain in the surgical wound and kneeling difficulty in their patients. Therefore, surgeons performing osteochondral transplantations and harvesting autografts from the knee should be aware of the potentially negative effect of clinical outcomes after surgery [30]. Therefore, Giannini et al. [11] chose to harvest cartilage from around the ankle joint rather than from the knee joint area. Their studies have reported that favourable clinical results were obtained when using the detached osteochondral fragments and talar neck cartilage as a source of cells for ACI in the ankle joint [11]. Giannini et al. [11, 12] reported that the AOFAS score increased from 32 to 90 in eight cases when detached fragments of cartilage were obtained from the talus cartilage defect site and used for chondrocyte culture in ACI. In addition, when Baums et al. [3] harvested 3.0-mm-diameter donor cartilage from the anterior chondral area of the talus for ACI, the AOFAS scores increased from 44 to 88, the HSS increased from 40 to 86, and the VAS decreased from 7.8 to 1.3. Matricali et al. [22] introduced an arthroscopic chondrocyte biopsy technique at the posteromedial rim of the talar dome by cadaveric study. In the current study, *the superior corner of the cuboid surface of the calcaneus* was used as the donor site, as favourable results had been achieved when excising old fractures of the anterior process of the calcaneus which include the cuboid surface of the calcaneus [8, 33, 36]. This study also showed that when the cuboid surface of the calcaneus was used as the donor site, it did not affect the patients’ daily activities or the function of their ankle joints, as noted at their final follow-up visits.

The current study evaluated the follow-up MRI findings and found that 80 % (28/35) showed subchondral plate irregularity and 50 % (3/6) of subchondral cysts disappeared without bone grafting. In the study by Nam et al. [27], 45 % (4/9) reported with focal defects compared with the level relative to the adjacent subchondral plate. Their results included four patients with an increase in cyst size (1 sandwich procedure, 3 ACI) and five with a decrease in cyst size (4 with sandwich procedure, 1 ACI). They recommended sandwich bone grafting for cystic lesions that may be attributed to elimination of the subchondral pain generated by the cyst itself. The subchondral plate irregularities seen in the current study were expected in patients who had the delaminating technique performed by drilling on the subchondral plate. However, in the current study it was shown that subchondral plate irregularity does not affect a patient’s ankle clinical score. Complete debridement of the cystic lesion was effective; however, the necessity for bone grafting was not essential.

The current study had several limitations, as it did not compare the gel-type ACI with other techniques like microfracture and osteochondral plug implantation. In addition, patients were enrolled with osteochondral lesions of 10 cm^2^ or less. However, the general consensus for the use of ACI is in treating larger-sized osteochondral lesions. Therefore, the superiority of ACI for larger-sized osteochondral lesion was not demonstrated here. However, this study has a relatively large number of amenable second-look arthroscopic data suggesting that the gel-type ACI procedure is potentially useful. Further study is required, using a randomized comparative study design, comparing between open and arthroscopic techniques, between different donor sites and a controlled study using the gel-type ACI procedure to treat larger-sized osteochondral lesions.

The clinical and arthroscopic results from this study demonstrate that using a mixed thrombin and fibrin gel form for chondrocyte matrix implantation may be a useful treatment for osteochondral lesions of the talus.

## Conclusions

Gel-type ACI using autologous chondrocytes of the cuboid surface of the calcaneus was effective for improving talar cartilage defects, was relatively easy to perform and did not induce morbidity at the donor site. Therefore, taken together, the results suggest that this implantation technique may be successful for treating an osteochondral lesion of the talus.
